# Impact of mergers and acquisitions on firms’ performance adjusted to business cycle fluctuations in China

**DOI:** 10.1371/journal.pone.0318024

**Published:** 2025-01-24

**Authors:** Yi Zhang, Tong Lin, Yuanbo Qiao

**Affiliations:** 1 School of Business Administration, Liaoning Technical University, Huludao, Liaoning, China; 2 School of Urban Economics and Public Administration, Capital University of Economics and Business, Beijing, China; 3 Institute for Studies in County Development, Shandong University, Qingdao, Shandong, China; 4 Qingdao Institute of Humanities and Social Sciences, Shandong University, Qingdao, Shandong, China; 5 Center for Yellow River Ecosystem Products, Shandong University, Qingdao, Shandong, China; Lund University, SWEDEN

## Abstract

This research mainly explored the effects of mergers and acquisitions (M&As) on the financial performance of Chinese listed companies and the determinants of post-M&A financial performance of mergers by incorporating adjustments for business cycle fluctuations. The research was divided into two parts. The first part applied data envelopment analysis (DEA) models for the calculation of the financial performance scores of mergers and non-mergers in six major sectors before and after M&As. Comparative analyses of financial performance trends between mergers and non-mergers in similar sectors revealed that M&As often decrease financial performance scores of mergers compared with non-mergers. The second part adopted regression analyses and robustness test to evaluate the effects of listing duration, financial leverage level, free cash flow and target type on post-M&A financial performance. The results showed that mergers’ sufficient listing duration, high financial leverages, adequate free cash flow and asset as target had significant positive impact on the post-M&A performance of mergers. Opposite to the free cash flow hypothesis, this study revealed that free cash flow is beneficial for mergers in China. These findings emphasized that lack of experience and managerial hubris are the primary factors contributing to the underperformance observed within the Chinese M&A market.

## 1 Introduction

Conventional metrics of financial performance, including earnings per share and return on assets, provide a constrained view of a company’s financial standing due to their singular focus [1]. To address this limitation, several scholars have employed Data Envelopment Analysis (DEA), a technique for assessing relative efficiency, to conduct a more detailed and comprehensive analysis of financial performance. By benchmarking an entity’s performance against a group of comparable peers, DEA adeptly captures the interactions among multiple ratios, synthesizing these insights into a unified relative efficiency score that offers a holistic evaluation [2]. The existing research that leverages DEA to evaluate the impact of mergers and acquisitions (M&As) on corporate financial performance, it is a common practice to use the annual financial outcomes of companies as comparable units [3]. However, this approach is difficult to distinguish whether the observed financial performance changes are attributable to the implementation of M&A strategy or to external factors such as business cycle fluctuations. This study adopted a novel methodology utilizing DEA to measure the relative efficiency shifts among industry peers, allowing for the isolation of business cycle impacts and a clearer understanding of how M&A activities intrinsically affect corporate financial performance.

Mergers and acquisitions (M&As) are among the most critical events in the lifecycle of an enterprise [4]; they can transform the business landscape and operational model of an enterprise through various mechanisms such as mergers, consolidations, and stock and asset acquisitions [5], enabling the acquiring company to expand its market scale, improve its production efficiency and cultivate competitive advantages [6]. Therefore, M&As play a key role in efficient resource allocation in the economy [7], which has attracted the attention of researchers for many years. However, the impact of M&As on corporate financial performance remains a highly contentious issue. Some research works have indicated that acquiring companies had proved successful, as various accounting measures had presented significant improvement [8–10]. On the other hand, some other studies have revealed that acquiring companies had not been successful and there had been remarkable declines in corporate financial performance after M&A [11–13]. These contradictory research findings are likely due to the neglect of accounting for the disturbances triggered by business cycle fluctuations. We were committed to tackling this issue by measuring the changes in financial performance scores of acquiring companies relative to their industry peers before and after M&As. By doing so, we aimed to mitigate the confounding effects of business cycle fluctuations and thereby precisely gauged the direct influence of M&As on corporate financial performance.

In the context of globalization, China has capitalized on its demographic dividend, leading to rapid advancements in foreign trade processing and related sectors [14]. This has expedited the industrialization of businesses and heightened the competitive landscape [15]. Consequently, a significant number of enterprises are seeking to augment their economies of scale and capture greater market share by engaging in M&As [16]. In 2022, Chinese A-share listed companies announced 6832 M&A transactions, with annual increase of 17.11%, and transaction size of about 2.8 trillion Chinese yuan with annual increase of 27.64% [17]. China has become one of the countries with the most intensive and extensive participation in M&As in the world [18,19]. Furthermore, the Chinese market is characterized by a multitude of family businesses, concentrated ownership, and a weak regulatory system [20]. Previous M&A research has primarily focused on developed markets, with emerging markets receiving less attention [21]. Numerous research findings necessitate further validation the context of China’s M&A market. Therefore, it is deemed crucial to examine the impact of M&A on the financial performance of Chinese companies, as well as the influencing factors, given the substantial wealth involved and the irreversible effects on all stakeholders, including target and acquiring entities, employees of both parties, and the entire supply chain.

The current scholars’ discussion on the impact of M&A on corporate financial performance has not sufficiently incorporated adjustments for business cycle fluctuations. However, Business cycles are common in any market economy and during these cyclical phases of an economy, enterprises experience peaks and troughs due to market demand and supply. During peak business seasons, companies achieve high sales and profits; however, when the tide turns, business entities suffer from volatility in sales, profitability, and cash flow [22–24]. Since the introduction of reform and opening-up policy, China’s economy has experienced the impacts of 1998 Asian economic crisis and 2008 global economic crisis during its growth process. Overall, the financial performance of Chinese listed companies was found to be consistent with fluctuations in Chinese business cycle [25]. Therefore, both corporate strategies and business cycle fluctuations have a significant impact on the financial data of Chinese listed companies. Moreover, the motivations and implementation experience behind M&As influence the post-M&A financial performance of firms. The obvious motive for M&A activities is to reap synergistic benefits, expand market share, and achieve rapid growth. However, some research in developed markets reveals underlying agency and hubris motives for M&As, such as free cash flow [26] and managerial hubris [27]. It remains to be examined whether these M&A motives, stemming from agency problems, are also present in China’s M&A market. Additionally, M&A is a complex capital operation that encompasses candidate selection, target valuation, and post-merger integration, representing a learning process for enhancing the acquisition process itself. The capital operation experience of the acquirer in a particular target country is subject to regional limitations [28]. Enterprises with an extended A-share listing duration are more likely to have rich experiences in capital operation and corporate governance in China’s capital market. It is yet to be examined whether these experiences foster enterprises in achieving superior domestic M&A outcomes. In light of this, we have chosen to examine mergers and non-mergers within similar sectors and employed DEA approach to calculate the shifts in relative performance scores of mergers before and after M&As. This methodology allowed us to explore the intrinsic impact of M&A on corporate financial performance by incorporating adjustments for business cycle fluctuations. Subsequently, this paper analyzed the impact of the listing duration of mergers, their asset-liability ratios, free cash flow, and target types on the short- and medium-term financial performance variations after M&As. This analysis aimed to examine the validity of the learning-by-doing theory, the free cash flow agency hypothesis and the managerial hubris hypothesis within the context of China’s M&A market.

The remainder of this paper is organized as follows. In Section 2: Literature review and hypothesis development, we introduce related literature and theories and build our central hypothesis. In Section 3: Research method and data source, we discuss the employed methodologies, variables, and data sources. In Section 4: Performance results and comparative analyses, we explore empirical results of M&A financial performance. In Section 5: Regression findings, we explain empirical results of post-M&A financial performance determinants. In Section 6: Robustness check, we conduct relevant robustness tests on independent variables. In Section 7: Conclusion, we summarize and conclude the paper.

## 2 Literature review and hypothesis development

### 2.1 Measurement of M&A financial performance

Empirical researchers have employed three prevailing approaches to evaluate the impacts of M&A transactions on corporate financial performance; i.e., financial ratios analysis [1], factor analysis [29] and DEA method [3]. DEA method is more objective in extracting overall financial performance scores compared to financial ratios analysis and factor analysis, since input and output weights for each DMU are calculated most favorable for that particular DMU rather than being set in advance. In DEA model, DMUs can be any set of entities converting comparable inputs into comparable outputs, such as companies operating in the same industry [30]. However, the existing research works have generally taken the financial performance of the acquiring company each year before and after M&A as DMUs for comparative analyses. For example, some research works have applied DEA models to analyze the impacts of M&A activities on the financial performance of Chinese A-share listed companies. Their findings indicated that M&As had significant positive effects on the financial performance of listed companies and with the passage of time after M&A, financial performance improvement showed steady upward trend [9,31]. However, other research works based on DEA method have demonstrated that the financial performance of Chinese A-share listed companies after M&As had not been effectively improved, but had decreased [32,33]. These research findings on M&A performance in China were contradictory, partly due to the effect of Chinese business cycle fluctuations [25]. In addition, inverse DEA has been applied in a recent research on M&As across various sectors such as banking, agriculture, and hospitality [34–36]. This approach is applied to determine optimal input or/and output levels, facilitating planned merger to meet some preset efficiency targets. It provides decision makers with a framework to perform more prudent evaluations during pre-M&A stage. This research primarily focused on the empirical comparison of the financial performance of Chinese listed companies before and after M&As; therefore, we employed standard DEA models to perform our research. By comparing financial performance of mergers and non-mergers in similar sectors, the influence of business cycle fluctuations was eradicated and the effect of M&A on the financial performance of corporates was restored, which was of great significance for exploring the effectiveness of M&A in China’s capital market.

### 2.2 Determinants of post-M&A financial performance

Regarding the factors influencing post-M&A financial performance, scholars have engaged in extensive discussions and developed numerous theories. These can be broadly categorized into two representative types: the motives behind M&A and the execution of M&A. Theories related to M&A motives include synergy theory, agency problems such as empire building, free cash flow agency hypothesis, managerial hubris hypothesis, and others. Synergy theory posits that the value of consolidated companies can exceed the combined values of the individual firms involved in the M&A, with synergies arising from sales, operational, investment, and managerial synergies [37]. Empire building theory elucidates the propensity of corporate management to pursue expansionary endeavors for personal benefit, which may not consistently serve the optimal interests of the company’s shareholders [38]. Since management’s compensation is often tied to the scale of the company, they are motivated to expand the company through M&A to increase their power and compensation [39]. Theories related to the execution of M&A include stakeholder theory, learning-by-doing theory and others. Stakeholder theory posits that corporate performance is shaped by a broader spectrum of stakeholders beyond just shareholders. In the context of M&A, considering the interests of employees, customers, suppliers, and communities is essential for enhancing the enterprise’s overall value. As a result, stakeholder interests, both internal and external, significantly influence M&A decisions and their outcomes [40]. Empirical investigations into the performance of M&As within China have shown inconsistent results, suggesting that synergistic goals are not always achieved after M&As [10–12]. Therefore, this paper further examined other underlying motives and execution of M&As, encompassing agency issues, managerial hubris, and corporate proficiency in capital operations, to evaluate their influence on post-M&A outcome.

#### 2.2.1 Experience

Learning-by-doing theory indicates that experience can form knowledge to improve productivity [41]. Acquisition process can be assumed as a learning process and the accumulation of experience can enhance the acquisition process itself, but this experience is limited to a particular target country [28]. Several research works on the cross-border M&As of Chinese listed companies have concluded that overseas operation and investment experiences of Chinese enterprises could effectively improve their cross-border M&A performance [42,43]. Furthermore, some research findings have indicated that, compared to newly established firms, companies with long establishment periods had richer management experience, knowledge accumulation, and talent reserves, which constituted the core competitiveness of enterprises and positively affected their M&A performance [44,45]. Since the M&A behaviors of Chinese A-share listed companies are regulated by China Securities Regulatory Commission, mature listed companies in Chinese capital market are more likely to have rich experience in capital operation and corporate governance, making it easier for them to achieve high M&A performance in compliance with regulatory requirements. In summary, we proposed Hypothesis 1:

H1: Sufficient listing duration of the merger is positively related to its post-M&A financial performance.

#### 2.2.2 Financial leverage level

Based on free cash flow agency hypothesis [26], agency conflicts among shareholders and managers becomes more severe when a company has a large amount of cash flow or its cash flow exceeds the requirements for paying dividends and investing in projects with positive net present values. To expand the scope of power or seek personal interests, managers prefer to invest excess cash into projects that cannot increase shareholder value and M&As are one of the main paths. Some research works on Chinese M&A market have also found that companies with high cash flow engage in excessive investment behaviors. Companies adopting high financial leverages have governance effects, which can constrain the self-interest behaviors of mergers, reduce their encroachment on shareholder interests, make managers more cautious in making M&A decisions, and improve the M&A performance of enterprises [46–48]. In summary, we proposed Hypothesis 2:

H2: High financial leverage level of the merger is positively related to its post-M&A financial performance.

#### 2.2.3 Type of target

Managerial hubris hypothesis indicates that hubristic executives tend to overestimate M&A synergies in valuation process, resulting in irrational increase in M&A prices [27]. M&A premium overpayment often leads to a series of subsequent risks and excess goodwill directly results from high-premium M&As [49]. Based on Accounting Standard for Business Enterprises No. 20 [50], in the case of a business combination not under the same control, the acquiring party shall recognize the difference between the acquisition cost and fair value of identifiable net assets obtained from acquired party as goodwill. According to Accounting Standard for Business Enterprises No. 8 [51], on the balance sheet date of enterprise, the goodwill formed by business combination and intangible assets with uncertain service life, regardless of whether there are impairment signs, should be evaluated for impairment annually. Some research results have shown that impairment loss of goodwill generated by corporate M&A resulted in decrease of post-M&A financial performance of acquiring companies [52,53]. Acquiring targets based on assets rather than equity is more likely to decrease the excess goodwill generated by managerial hubris by enhancing asset identifiability. He [54] and Wu [55] indicated that asset-oriented M&As performed better in Chinese M&A market compared to equity-oriented M&As. In summary, we proposed Hypothesis 3:

H3: Acquiring targets based on assets rather than equity can result in superior post-M&A financial performance for the merger.

## 3 Research method and data source

### 3.1 Sample selection and data source

This research adopted the announcement date of M&A completion between 2017 and 2019 to select mergers and corresponding non-mergers. M&A and financial data were obtained from China Stock Market & Accounting Research (CSMAR) database. Our research sample, including mergers and non-mergers in similar sectors, the following five selection criteria were considered: (1) both mergers and non-mergers were among the listed companies on Shanghai and Shenzhen stock exchanges to ensure access to publicly available financial data; (2) for mergers with multiple M&As in one year, the largest one was selected as a sample; (3) the total assets and operating revenue of non-mergers were between the maximum and minimum values of mergers in similar sectors, to guarantee the comparability of mergers and non-mergers; (4) quantity variance between mergers and non-mergers in similar sectors should not be too large when calculating financial performance scores among peers; (5) mergers and non-mergers with missing data were eliminated. Consequently, 616 mergers and 2996 non-mergers in six major sectors with the highest completion volumes of M&A transactions, including chemicals, pharmaceuticals, special equipment, electrical machinery, computer communication and software information sectors, were selected as the final sample.

Table 1 presents that M&A transaction sample in six major sectors accounted for over 40% of the total completion volume of M&A deals each year. The table shows that they were almost evenly distributed across the study period. Table 2 presents the proportion of mergers to non-mergers in six major sectors, about 20%, and it was found that the proportions changed among different sectors were very narrow.

**Table 1:** Sample distribution of M&A transactions.

Sector	M&A year		
	2017	2018	2019
Chemicals	36	35	44
Pharmaceuticals	27	23	23
Special Equipment	26	36	23
Electrical Machinery	40	32	29
Computer Communication	56	48	35
Software Information	39	35	29
Sum of annual sampled M&A deals	224	209	183
Sum of annual M&A completion deals	529	458	411
Proportion of selected samples to M&A deals	42.34%	45.63%	44.53%

This table presents the sample distribution of M&A transactions by year and industry, comprising 616 completed Chinese domestic M&As in six major sectors with the highest M&A completion volume between 2017 and 2019.

**Table 2:** Sample distribution of mergers and non-mergers.

Sector	Group	M&A year			Sum of mergers and non-mergers	Proportion of mergers to non-mergers
		2017	2018	2019		
Chemicals	Mergers	36	35	44	115	24.36%
	Non-mergers	131	161	180	472	
Pharmaceuticals	Mergers	27	23	23	73	14.04%
	Non-mergers	155	170	195	520	
Special Equipment	Mergers	26	36	23	85	21.96%
	Non-mergers	114	120	153	387	
Electrical Machinery	Mergers	40	32	29	101	21.04%
	Non-mergers	145	181	154	480	
Computer Communication	Mergers	56	48	35	139	19.52%
	Non-mergers	201	251	260	712	
Software Information	Mergers	39	35	29	103	24.24%
	Non-mergers	127	133	165	425	

This table presents the sample distribution of mergers and non-mergers by year and industry, comprising 616 mergers and 2996 non-mergers in six major sectors with the highest M&A completion volume between 2017 and 2019.

### 3.2 Measurement of variables

#### 3.2.1 Financial performance score

In the first part of the comparative analysis, based on the existing literature on measuring financial performance scores of enterprises using DEA method, this research identified a set of input and output indicators which were particularly relevant to the evaluation of the financial performance of corporates [9,56,57]. Input indicators included total assets, operating costs, taxes and surcharges, and operating expenses. Total assets were applied for examining the allocation of total resources owned by the company. Operating costs, taxes and surcharges were related inputs incurred by companies to generate operating revenue. Operating expenses examined various fees incurred by a company in its daily operation. Output indicators covered operating revenue and net profit. Operating revenue reflected the income generated by daily business activities of the company. Net profit demonstrated the overall profitability of the company. The selected input and output indicators could objectively evaluate the input-output levels of the company and no strong linear correlation was found between them. Table 3 summarizes the relevant indicators.

**Table 3:** Specification of inputs and outputs.

Indicator	Description
Total assets	The value of all assets owned or controlled by a company.
Operating costs, taxes and surcharges	The annual cost of selling goods or providing labor, related taxes, and fees by a company.
Operating expenses	The annual expenditure of sales, management, R&D, and financing by a company.
Operating revenue	The annual revenue is the sale of goods or the provision of labor by a company.
Net profit	The annual value of income after taxes by a company.

#### 3.2.2 Dependent variables

In the second part of regression analyses, variations in the short- and medium-term financial performance scores of mergers after M&As were adopted as dependent variables. Variations in short-term financial performance scores of mergers after M&As calculated by constant returns to scale (CRS) and variable returns to scale (VRS) models were represented by the two variables of ΔSTPcrs and ΔSTPvrs. Variations in medium-term financial performance scores of mergers after M&As calculated by CRS and VRS models were represented by the two variables of ΔMTPcrs and ΔMTPvrs.

#### 3.2.3 Independent variables

Experience denoted the listing duration of a merger in Chinese A-share capital market. Under Rules Governing the Listing of Stocks [58,59], the controlling shareholder of an A-share listed company shall not transfer or entrust others to manage its shares within three years from the initial public offering (IPO) date. This policy aimed to restrain the diligence of decision-makers and enable companies to better adapt to the governance and capital operation rules of listed companies. Therefore, we concluded that if the listing duration of a merger was ≥ 3 years, it was a mature listed company and took 1; otherwise, it took 0 and was considered as a young listed company.

Debt denoted the asset-liability ratio of a merger, which was an indicator to measure its financial leverage level. Referring to the findings of Li and Li [48], Zhang and Liu [47], and Zhao et al. [46], we calculated asset-liability ratio as the book value of total liabilities/ book value of total assets ×100% on the balance sheet date of the year before the announcement of M&A completion.

Type denoted target type in an M&A transaction and we found that if the target was an asset, it took 1; otherwise, it took 0 for equity.

#### 3.2.4 Control variables

Control variables in our regression analyses were related to acquirer- and M&A deal-specific characteristics derived from literature [60–62] and included state-owned enterprise (SOE) dummy, the proportion held by the largest shareholder (Top1), price earnings ratio (PE), industry relatedness (Goal) dummy, mode of payment (Payment) dummy, non-cross city M&A (Non-cross) dummy, non-related party transaction (Non-related) dummy, material asset reorganization deal (Major) dummy, and the natural logarithm of deal price (Price), all of which could affect variations in the short- and medium-term financial performance scores of mergers after M&As. Furthermore, we included fixed effects of industry of merger (Industry) and announcement year of M&A completion (M&A year) to control for cross-industry and time-variant impacts on variations in the short- and medium-term financial performance scores of mergers after M&As. Table 4 summarizes the definitions of relevant variables.

**Table 4:** Variable definitions.

Category	Variable	Definitions
Dependent Variables	ΔSTPcrs	Variations of short-term financial performance score of the merger after M&A, calculated as the difference between the average financial performance score of two years after M&A (t to t+1) and the financial performance score of one year before M&A (t-1) based on CRS model.
	ΔSTPvrs	Variations of short-term financial performance score of the merger after M&A, calculated as the difference between the average financial performance score of two years after M&A (t to t+1) and the financial performance score of one year before M&A (t-1) based on VRS model.
	ΔMTPcrs	Variations of medium-term financial performance score of the merger after M&A, calculated as the difference between the average financial performance score of two years after M&A (t+2 to t+3) and the financial performance score of one year before M&A (t-1) based on CRS model.
	ΔMTPvrs	Variations of medium-term financial performance score of the merger after M&A, calculated as the difference between the average financial performance score of two years after M&A (t+2 to t+3) and the financial performance score of one year before M&A (t-1) based on VRS model.
Independent Variables	Experience	The dummy variable of whether the merger is a mature listed company, calculated as (announcement date of M&A completion–date of initial public offerings)/365, if its listing duration is ≥ 3 years, it is a mature listed company and takes 1; otherwise, it takes 0 and is a young listed company.
	Debt	The asset-liability ratio of the merger is calculated as the book value of total liabilities/ book value of total assets×100% on the balance sheet date of the year before the announcement of M&A completion.
	Type	The dummy variable of the target type, if the target is an asset, it takes 1; otherwise, it takes 0 for equity.
Control Variables	SOE	The dummy variable of whether the merger is a state-owned enterprise, if a state-owned enterprise it takes 1; otherwise, it takes 0.
	Top1	The shareholding ratio of the largest shareholder of the merger on the balance sheet date of the year before the announcement of M&A completion.
	PE	The natural logarithm of price-earnings ratio of the merger on the balance sheet date of the year before the announcement of M&A completion.
	Goal	The dummy variable of industry relatedness, if it is a horizontal M&A, it takes 1; if it is a vertical M&A, it takes 2; otherwise, it takes 3.
	Payment	The dummy variable of the mode of payment, if paid by cash, it takes 1; otherwise, it takes 0.
	Non-cross	The dummy variable of whether it is a non-cross city M&A, if it is a non-cross city M&A, it takes 1; otherwise, it takes 0.
	Non-related	The dummy variable of whether it is a non-related party transaction, if it is a non-related party transaction, it takes 1; otherwise, it takes 0.
	Major	The dummy variable of whether M&A is a material asset reorganization deal, if it is a material asset reorganization deal, it takes 1; otherwise, it takes 0.
	Price	The natural logarithm of deal price paid by the merger in a M&A transaction.
	Industry	Industry of merger.
	M&A year	The announcement year of M&A completion.

### 3.3 Methodology

#### 3.3.1 DEA approach

DEA is a non-parametric method for measuring efficiency by a linear programming model, which does not need to know the specific form of production function, but only needs to obtain the input-output data. DEA model can incorporate multiple inputs and outputs for the identification of an efficient frontier through all DMUs and measurement of the distances of inefficient DMUs from the frontier. These DMUs could be business units, such as companies operating in similar industries [30], and the efficiency scores of DMUs vary in the range of 0 to 1. There are two widely applied DEA models, namely CRS model [63] and VRS model [64]. In essence, VRS model considers scale efficiency (SE) according to CRS model, decomposing overall technical efficiency (OTE) in CRS model into two parts of SE and pure technical efficiency (PTE). The relationship between technical efficiencies based on CRS and VRS models is as follows:

$$\mathrm{Scale}\;\mathrm{efficiency}\;(\mathrm{SE})=\frac{\mathrm{Overall}\;\mathrm{technical}\;\mathrm{efficiency}\;(\mathrm{under}\;\mathrm{CRS})}{\mathrm{Pure}\;\mathrm{technical}\;\mathrm{efficiency}\;(\mathrm{under}\;\mathrm{VRS})}$$ (1)

OTE is effective only if both SE and PTE are effective. CRS model is designed based on the following program:

$$Min\;\theta_{0}$$ (2)

s.t.

$$\overset{n}{\underset{j=1}{\sum }}\lambda_{0j}y_{rj}\geq y_{r0}\;r=1,2,\ldots ,s$$ (3)

$$\overset{n}{\underset{j=1}{\sum }}\lambda_{0j}x_{ij}\leq \theta_{0}x_{i0}\;i=1,2,\ldots ,m$$ (4)

$$\lambda_{0j}\geq 0\;j=1,2,\ldots ,n$$ (5)

where *θ*_0_ is OTE, *y*_*r*0_ is the r^th^ output of *DMU*_0_, *x*_*i*0_ is the i^th^ input of *DMU*_0_, *y*_*rj*_ is the r^th^ output concerning the unit of reference j, *x*_*ij*_ is the i^th^ input concerning the unit of reference j, *λ*_0*j*_ is the weight of reference unit j, *s* is the number of outputs, *m* is the number of inputs, and *n* is the number of DMUs. Compared with CRS model, VRS model only adds one constraint condition $\sum_{j=1}^{n}\lambda_{0j}=1$, limiting the value of *λ*_0_; *θ*_0_ is PTE in this condition.

DEA models can be either input-oriented or output-oriented. Input-oriented models seek to minimize inputs for a given output level, while output-oriented models aim to maximize outputs with a fixed input level. Typically, input-oriented models posit that DMUs have little control over their outputs, unlike output-oriented models, which assume direct control. The listed companies that formed the basis of this research had limited control over outputs such as sales and profits. However, they possess full discretion over inputs, including asset investment, operating costs, and expenses. Based on this reality and referring to pertinent literature [65–67], this research adopted input-oriented DEA models. In practice, the concept of constant returns to scale is an idealized assumption. However, some research works have indicated that, due to factors such as R&D, companies in pharmaceuticals, hospitality, and manufacturing sectors exhibited constant returns to scale over a certain period [66,68,69]. Whether enterprises sustain constant returns to scale before and after M&As over a certain period is a contentious issue [65,67]. Drawing on previous research works [70–72], this study employed both the CRS and VRS models using the DEA Solver Pro 5.0 software for performance analyses. CRS model provided a fundamental analysis, with VRS model serving as robustness test method, ensuring the reliability of the findings through cross-validation.

#### 3.3.2 Multivariate regression

We utilized multivariate regression analyses with the Stata/SE 15.1 software to investigate the association between listing duration of mergers, financial leverage levels of mergers, target type and variations in short- and medium-term financial performance scores of mergers after M&As. For multivariate analyses, we performed full sample robust regression in model (6) and subsample robust regression in model (7), as follows:

$$\Delta Performance_{i}^{1}=\beta_{0}+\beta_{1}Experience_{i}+\beta_{2}Debt_{i}+\beta_{3}Type_{i}+\sum Control+\sum Ind+\sum Year+\varepsilon_{i}$$ (6)

$$\Delta Performance_{i}^{2}=\beta_{0}+\beta_{1}Experience_{i}+\beta_{2}Debt_{i}+\beta_{3}Type_{i}+\sum Control+\sum Year+\varepsilon_{i}$$ (7)

In model (6), Δ*Performance*^1^ represents ΔSTPcrs, ΔSTPvrs, ΔMTPcrs and ΔMTPvrs, which denote variations in short- or medium-term financial performance scores of mergers after M&As and is calculated as the difference between post-M&A and pre-M&A financial performance scores of mergers. The main independent variables were listing duration of mergers, asset-liability ratios, and target type. Furthermore, we included the control variables discussed in Control variables subsection in the regressions, as well as the effects of industry and M&A year fixed. In model (7), Δ*Performance*^2^ represents ΔSTPcrs, ΔSTPvrs, ΔMTPcrs and ΔMTPvrs, which describe the variations of short- or medium-term financial performance scores of mergers after M&As in each major sector. We kept independent variables, control variables, and M&A year fixed effect unchanged. To enhance the robustness of regression results, all continuous variables in regression analyses for both the full sample and the subsample were processed by Winsor tailing up and down at the 1% level.

## 4 Performance results and comparative analyses

### 4.1 Descriptive statistics of inputs and outputs

Table 5 summarizes descriptive statistics for the inputs and outputs of mergers and corresponding non-mergers before and after M&As. The average values and volatilities of inputs and outputs in mergers were higher than those of non-mergers during research period. Mergers increased their inputs and outputs in post-M&A years, as measured by total assets, operating costs, taxes and surcharges, operating expenses, operating revenue, and net profit. However, non-mergers also increased their inputs and outputs in the same period. Therefore, the crucial question is whether the financial performance of mergers after M&As is improved compared to non-mergers, as measured by the financial performance scores of DEA models between mergers and non-mergers in similar sectors.

**Table 5:** Descriptive statistics of inputs and outputs.

Group	Indicator	Pre-M&A year (t-1)	M&A year (t)	Post-M&A year (t+1)	Post-M&A year (t+2)	Post-M&A year (t+3)
Mergers	Total assets	5478	7105	7813	8549	9542
		(14480)	(17230)	(19760)	(21000)	(23880)
	Operating costs, taxes and surcharges	2206	3007	3386	3658	4052
		(7021)	(9164)	(10450)	(10760)	(11690)
	Operating expenses	558	759	845	873	905
		(1585)	(2009)	(2045)	(1801)	(1722)
	Operating revenue	3062	4136	4633	4981	5420
		(9515)	(12390)	(13820)	(13980)	(14650)
	Net profit	286	338	284	355	361
		(1285)	(1550)	(1686)	(1849)	(1732)
Non-mergers	Total assets	4565	4935	5397	5991	6760
		(6362)	(6742)	(7370)	(8385)	(9737)
	Operating costs, taxes and surcharges	1852	2042	2217	2534	2935
		(3619)	(3810)	(4081)	(4859)	(5841)
	Operating expenses	527	611	666	719	755
		(939)	(1063)	(1115)	(1225)	(1286)
	Operating revenue	2602	2880	3121	3549	4065
		(4514)	(4805)	(5114)	(6104)	(7276)
	Net profit	184	155	164	231	315
		(491)	(1047)	(684)	(887)	(1037)

This table presents descriptive statistics for the mean and standard deviation of inputs and outputs of mergers and non-mergers in six major sectors before and after M&As (in CNY million).

### 4.2 Merger and non-merger financial performance

**Fig 1:**
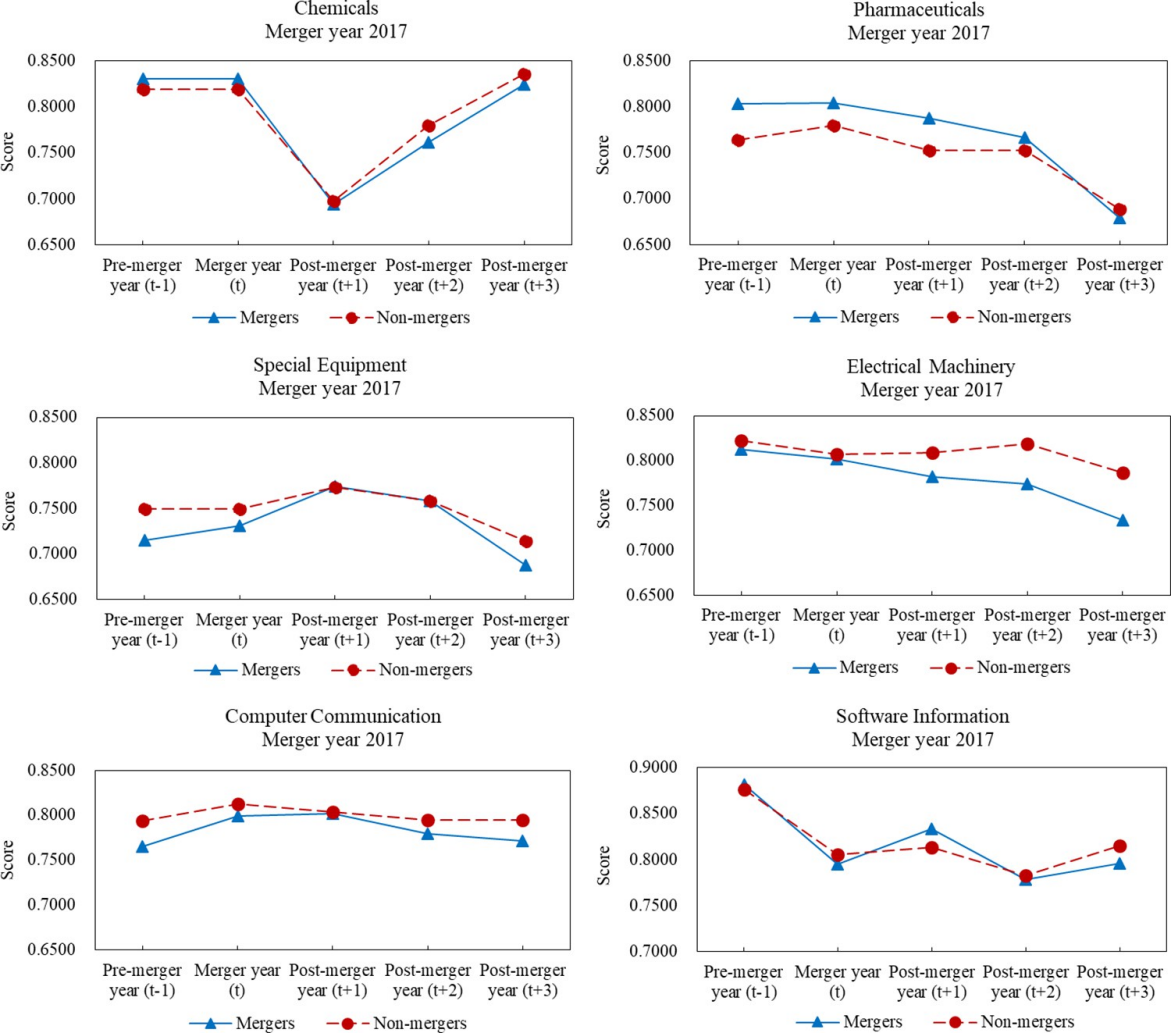
Comparative analyses of performance scores between mergers and non-mergers in 2017.

Fig 1 illustrates that, according to CRS model, the mean financial performance scores of mergers and non-mergers in most sectors in three years after M&As were decreased compared to those of one year before M&As, with only chemicals and computer communication sectors remaining almost at their original levels. In addition to special equipment and computer communication sectors, mean financial performance scores of mergers were decreased more than non-mergers after M&As. Variations in financial performance showed various trends across six major sectors. For pharmaceuticals, special equipment, and computer communication sectors, fluctuations were first increased and then decreased. However, for chemicals and software information sectors, fluctuations were first downward and then upward. Furthermore, the mean financial performance scores of two sets of samples in electrical machinery sector showed an almost continuous downward trend. Similar results obtained from VRS model can be seen within S1 Fig.

**Fig 2:**
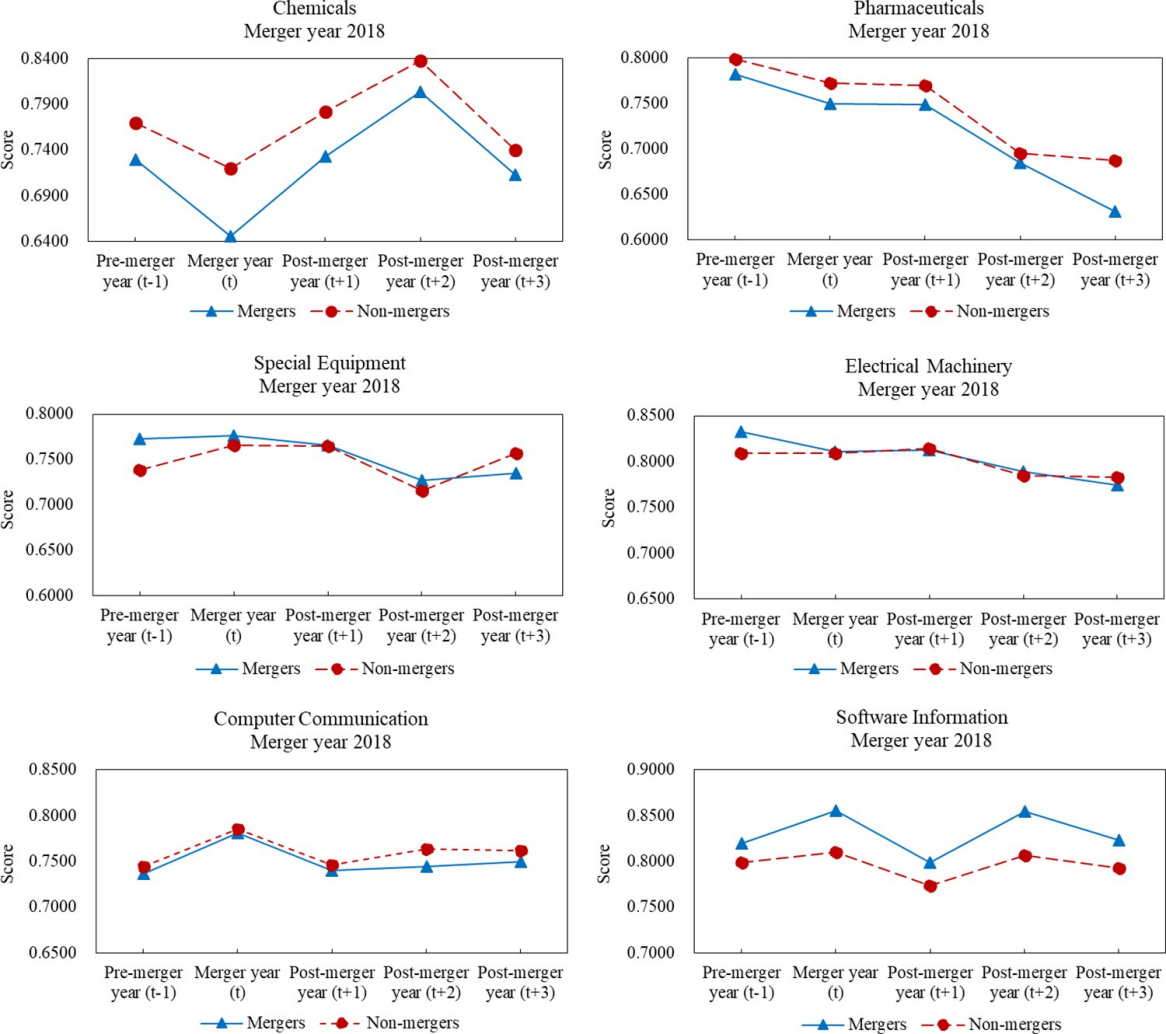
Comparative analyses of performance scores between mergers and non-mergers in 2018.

As illustrated in Fig 2, according to CRS model, the mean financial performance scores of mergers and non-mergers three years after M&As in most sectors were lower than those of one year before M&As and only computer communication sector maintained its initial level. Besides chemicals and software information sectors, mean financial performance scores of mergers became worse than those of non-mergers after M&As. Variations in financial performance exhibited different trends across six major sectors. For special equipment, computer communication and software information sectors, financial performance trends were first increased and then decreased, but for chemicals sector, the fluctuation was first downward and then upward and for pharmaceuticals and electrical machinery sectors, the mean financial performance scores of two sets of samples were almost continuously declined. Similar results from VRS model can be seen within S2 Fig.

**Fig 3:**
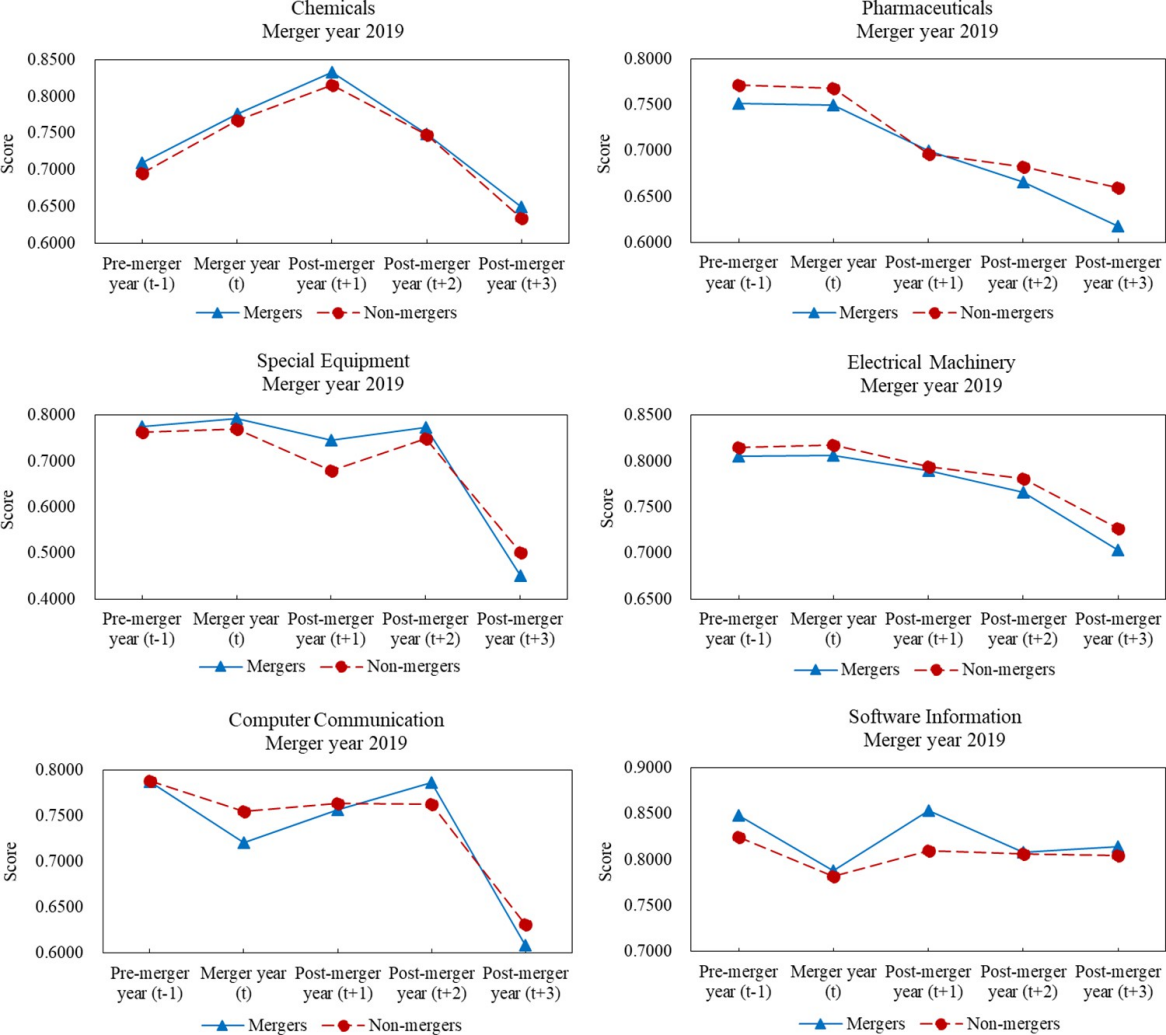
Comparative analyses of performance scores between mergers and non-mergers in 2019.

Fig 3 shows that according to CRS model, the mean financial performance scores of mergers and non-mergers in three years after M&As were decreased compared to those of one year before M&As and most sectors experienced significant declines. Besides chemicals sector, the mean financial performance scores of mergers were decreased more than those of non-mergers after M&As. Fluctuations of financial performance presented different forms in six major sectors. For chemicals, special equipment, and electrical machinery sectors, the financial performance trends were first increased and then decreased, but for software information sector, fluctuations were first downward and then upward. For pharmaceuticals and computer communication sectors, the mean financial performance scores of the two sets of samples showed an almost continuous downward trend. Similar results obtained from VRS model can be seen within S3 Fig.

Based on the financial performance trends of merger samples and corresponding non-merger samples from 2017 to 2019, M&A transactions did not improve the financial performance of mergers, as measured by financial performance scores according to CRS and VRS models. Based on the comparative analyses of the two sets of samples in six major sectors, M&As tended to result in poor financial performance for mergers compared to non-mergers. In most cases, the mean financial performance scores of mergers in pharmaceuticals, special equipment, and electrical machinery sectors were higher than those of non-mergers one year before M&As. However, the mean financial performance scores of mergers were obviously lower than those of non-mergers three years after M&As. In addition, the mean financial performance scores of mergers in software information sector were higher than those of non-mergers one year before M&As. However, in most cases, score gaps between mergers and non-mergers were obviously narrowed three years after M&As.

To test whether there were significant differences in mean financial performance scores between mergers and non-mergers before and after M&As, a paired sample t-test was carried out and the obtained results are summarized in Table 6. As could be seen from the table, the mean financial performance scores of mergers before M&As obtained from VRS model for pharmaceuticals, electrical machinery and software information sectors were significantly higher than those of corresponding non-mergers, with score differences of 0.0277 (p < 0.1), 0.0199 (p < 0.1) and 0.0286 (p < 0.05), respectively. However, after M&As, the mean financial performance scores of mergers in these three sectors were decreased and only software information sector maintained a significant score difference of 0.0179 (p < 0.1) between mergers and non-mergers. Numerical direction of score differences calculated based on CRS model was consistent with those of VRS model, but there were no statistically significant differences between the two sample sets. The results of paired sample t-test between mergers and non-mergers further confirmed observation findings in comparative analysis of performance trends.

**Table 6:** Results of paired sample t-test between mergers and non-mergers.

Sector	Indicator	Pre-M&A mean scores under CRS model	Pre-M&A mean scores under VRS model	Post-M&A mean scores under CRS model	Post-M&A mean scores under VRS model
Chemicals	Mergers	0.7534	0.8171	0.7515	0.7985
	Non-mergers	0.7550	0.8147	0.7627	0.8122
	Difference	-0.0015	0.0024	-0.0112	-0.0137
	P-value	0.9073	0.8499	0.2913	0.1857
Pharmaceuticals	Mergers	0.7804	0.8457	0.7179	0.7814
	Non-mergers	0.7783	0.8180	0.7239	0.7729
	Difference	0.0020	0.0277*	-0.0060	0.0085
	P-value	0.8892	0.0640	0.6833	0.5673
Special Equipment	Mergers	0.7557	0.8327	0.7305	0.7880
	Non-mergers	0.7518	0.8103	0.7202	0.7886
	Difference	0.0039	0.0224	0.0103	-0.0005
	P-value	0.7941	0.1653	0.4738	0.9691
Electrical Machinery	Mergers	0.8168	0.8722	0.7785	0.8283
	Non-mergers	0.8150	0.8523	0.7943	0.8420
	Difference	0.0018	0.0199*	-0.0158	-0.0136
	P-value	0.8659	0.0746	0.1278	0.1991
Computer Communication	Mergers	0.7609	0.8202	0.7585	0.8002
	Non-mergers	0.7744	0.8139	0.7615	0.8093
	Difference	-0.0135	0.0063	-0.0030	-0.0092
	P-value	0.2176	0.5676	0.7760	0.3658
Software Information	Mergers	0.8510	0.8914	0.8160	0.8624
	Non-mergers	0.8321	0.8628	0.8001	0.8446
	Difference	0.0190	0.0286**	0.0159	0.0179*
	P-value	0.1276	0.0155	0.1407	0.0919

Statistical significance at 10%, 5% and 1% confidence levels are indicated by

*, **, and ***, respectively.

### 4.3 Pre- and post-M&A financial performance

To test whether mean financial performance scores of mergers in short- and medium-terms after M&As were significantly different from those before M&As, a paired sample t-test was performed and the obtained results are presented in Table 7. Financial performance of post-M&A short-term was calculated as the average financial performance score two years after M&A (t to t+1) and that for post-M&A medium-term was obtained as the average financial performance score two years after M&A (t+2 to t+3). For pharmaceuticals, electrical machinery, and software information sectors using CRS model, the mean financial performance scores of mergers before M&As were significantly higher than those after M&As. Differences in scores between before and short-term after M&A were 0.0214 (p < 0.05), 0.0168 (p < 0.05), 0.0305 (p < 0.01), and those between before and medium-term after M&A were 0.1035 (p < 0.01), 0.0598 (p < 0.01), 0.0394 (p < 0.01), which indicated that financial performance of mergers in these three sectors continued to deteriorate from short-term to medium-term after M&As. For special equipment sector according to CRS model, the difference in scores between before and short-term after M&A was insignificant, but those between before and medium-term after M&A was 0.0595 (p < 0.01). Similar results were also obtained from VRS model. The t-test results comparing the relative performance scores of mergers among peers before and after M&As indicate that, across most sectors, M&As often led to a decline in corporate financial performance. These findings are supported by prior research, using the annual financial performance of mergers before and after M&As as comparable units, which found that the financial performance of companies deteriorated in the short to medium term following M&As [13,56]. There are also opposite findings that M&As have led to a steady improvement in the financial performance of mergers [3,9]. However, these prior studies did not account for business cycle fluctuations in their analyses.

**Table 7:** Results of paired sample t-test before and after M&A.

Sector	Indicator	Mean scores under CRS model	Mean scores under VRS model
Chemicals	Pre-M&A	0.7534	0.8171
	Post-M&A (short-term)	0.7562	0.8034
	Pre-and post-M&A difference (short-term)	-0.0028	0.0137
	P-value (short-term)	0.7774	0.2122
	Post-M&A (medium-term)	0.7467	0.7935
	Pre- and post-M&A difference (medium-term)	0.0066	0.0236**
	P-value (medium-term)	0.5308	0.0460
Pharmaceuticals	Pre-M&A	0.7804	0.8457
	Post-M&A (short-term)	0.7589	0.8186
	Pre- and post-M&A difference (short-term)	0.0214**	0.0271**
	P-value (short-term)	0.0488	0.0190
	Post-M&A (medium-term)	0.6768	0.7443
	Pre- and post-M&A difference (medium-term)	0.1035***	0.1014***
	P-value (medium-term)	0.0000	0.0000
Special Equipment	Pre-M&A	0.7557	0.8327
	Post-M&A (short-term)	0.7648	0.8219
	Pre- and post-M&A difference (short-term)	-0.0091	0.0108
	P-value (short-term)	0.4215	0.3505
	Post-M&A (medium-term)	0.6963	0.7541
	Pre- and post-M&A difference (medium-term)	0.0595***	0.0785***
	P-value (medium-term)	0.0002	0.0000
Electrical Machinery	Pre-M&A	0.8168	0.8722
	Post-M&A (short-term)	0.8000	0.8443
	Pre- and post-M&A difference (short-term)	0.0168**	0.0278***
	P-value (short-term)	0.0427	0.0008
	Post-M&A (medium-term)	0.7570	0.8123
	Pre- and post-M&A difference (medium-term)	0.0598***	0.0598***
	P-value (medium-term)	0.0000	0.0000
Computer Communication	Pre-M&A	0.7609	0.8202
	Post-M&A (short-term)	0.7711	0.8134
	Pre- and post-M&A difference (short-term)	-0.0102	0.0069
	P-value (short-term)	0.1858	0.4426
	Post-M&A (medium-term)	0.7458	0.7870
	Pre- and post-M&A difference (medium-term)	0.0151	0.0333**
	P-value (medium-term)	0.1486	0.0020
Software Information	Pre-M&A	0.8510	0.8914
	Post-M&A (short-term)	0.8205	0.8680
	Pre- and post-M&A difference (short-term)	0.0305***	0.0234***
	P-value (short-term)	0.0033	0.0081
	Post-M&A (medium-term)	0.8116	0.8568
	Pre- and post-M&A difference (medium-term)	0.0394***	0.0346***
	P-value (medium-term)	0.0022	0.0030

Statistical significance at 10%, 5% and 1% confidence levels are indicated by

*, **, and ***, respectively.

## 5 Regression findings

### 5.1 Descriptive statistics of variables

Table 8 summarizes the descriptive statistics of regression variables. According to CRS model, average variation in the short-term financial performance scores of mergers after M&As was -0.0067, the minimum score variation was -0.2667, and maximum shift in score was 0.2629. Accordingly, average variation in medium-term financial performance scores of mergers after M&As was -0.0174, minimum score variation was -0.3114, and maximum score variation was 0.2449. These figures indicated that the financial performance of mergers was continuously declined from short-term to medium-term after M&As. The results obtained from VRS model were similar to those obtained from CRS model. The proportion of mergers with listing duration of three years or more was about 70%, indicating that most mergers had mature understanding from capital operation and corporate governance in China’s capital market. The average asset-liability ratio of mergers was about 35%, with minimum and maximum levels of 7% and 82%, indicating that financial leverage level was significantly different among different companies. Assets as targets of M&As accounted for only 4% and others were equities. Similar to Zhang et al. [7], we found that cash payments accounted for about 74% of M&A transactions and low proportion of share payments was due to the prudent regulatory approval mechanism of China Securities Regulatory Commission for listed companies to issue shares to purchase targets. We use variance inflation factors (VIF) to test whether there is multicolinearity problem, the VIF were consistently under 3, suggesting that multicollinearity does not pose a significant concern for our analysis.

**Table 8:** Descriptive statistics of variables.

Variable	Obs.	Mean	Std. Dev.	Min.	Max.
ΔSTPcrs	616	-0.0067	0.0943	-0.2667	0.2629
ΔSTPvrs	616	-0.0174	0.0956	-0.3114	0.2449
ΔMTPcrs	616	-0.0413	0.1222	-0.3718	0.2662
ΔMTPvrs	616	-0.0501	0.1236	-0.3931	0.2578
Experience	616	0.70	0.46	0	1
Debt	616	0.35	0.18	0.07	0.82
Type	616	0.04	0.20	0	1
SOE	616	0.22	0.42	0	1
Top1	616	0.31	0.13	0.05	0.78
PE	616	4.05	0.91	2.06	7.18
Goal	616	2.16	0.63	1	3
Payment	616	0.74	0.44	0	1
Non-cross	616	0.28	0.45	0	1
Non-related	616	0.67	0.47	0	1
Major	616	0.23	0.42	0	1
Price	616	19.03	1.80	14.22	23.33

This table reports descriptive statistics of ΔSTPcrs, ΔSTPvrs, ΔMTPcrs, ΔMTPvrs, Experience, Debt, Type and control variables for mergers in six major sectors between 2017 and 2019.

### 5.2 Regression result analysis and hypothesis testing

Table 9 summarizes full sample robust regression results to evaluate all hypotheses. As presented in column (1), based on CRS model, the coefficients for Experience, Debt, Type associated with post-M&A short-term financial performance variation were 0.019, 0.067, and 0.034, respectively, which were significantly positively correlated at 5%, 1% and 10% levels. Column (3) indicates that according to CRS model, the coefficients for Experience, Debt, Type associated with post-M&A medium-term financial performance variation were 0.029, 0.080, and 0.041, respectively, showing significant positive correlation at 1%, 1%, and 10% levels. Another noteworthy point is that the dummy variable for 2019 M&A year had a significant positive effect on short-term post-M&A financial performance of mergers, but a significant negative effect on their medium-term performance. A plausible explanation for this discrepancy is that companies that completed M&As in 2019, shortly after the COVID-19 pandemic, experienced an inventory surge due to expanded operational scale, which mitigated the negative impact of pandemic-related disruptions on sales. However, as inventories dwindled, the financial performance of these companies, which had incurred increased labor costs due to M&As, became more severely affected by pandemic-induced disruptions. Besides, the significance of the F-statistics shows that the regression can explain the variance of performance to some extent. We also obtained similar results in columns (2) and (4) using VRS model. These results suggested that mergers had sufficient listing durations, high financial leverage levels and asset as target could effectively improve the post-M&A financial performance of mergers, and the effects of these factors became more pronounced with time; therefore, all hypotheses were supported. Similarly, prior studies employed the age of acquirer as a proxy for capital operation experience, demonstrating a significant positive correlation with post-M&A financial performance [44,45]. Furthermore, earlier scholars also confirmed that highly leveraged companies achieve superior post-M&A financial outcome, and they proposed that high leverage can mitigate managerial misuse of free cash flow [46]. However, companies with high leverage may aim to prevent managerial misuse of cash flow or leverage tax shield benefits. Thereby, the existence of free cash flow agency issue in Chinese M&A market necessitates robustness test using free cash flow as a proxy. Additionally, asset-oriented M&As showed to outperform equity-oriented ones in terms of financial performance, aligning with previous research findings [54,55].

**Table 9:** Full sample robust regression results of Experience, Debt, Type on post-M&A financial performance variation.

Variable	(1)	(2)	(3)	(4)
	ΔSTPcrs	ΔSTPvrs	ΔMTPcrs	ΔMTPvrs
Experience	0.019**	0.035***	0.029***	0.045***
	(2.31)	(4.09)	(2.72)	(4.09)
Debt	0.067***	0.068***	0.080***	0.073**
	(2.71)	(2.68)	(2.70)	(2.26)
Type	0.034*	0.039**	0.041*	0.056***
	(1.84)	(2.46)	(1.91)	(2.89)
SOE	0.003	0.016*	0.000	0.002
	(0.39)	(1.79)	(0.01)	(0.17)
Top1	0.027	0.022	0.012	0.015
	(0.92)	(0.80)	(0.35)	(0.44)
PE	0.017***	0.006	0.024***	0.014*
	(2.93)	(1.09)	(3.42)	(1.94)
Goal (vertical)	-0.009	-0.018	-0.005	-0.005
	(-0.85)	(-1.43)	(-0.35)	(-0.33)
Goal (others)	-0.004	-0.014	0.004	-0.005
	(-0.33)	(-1.06)	(0.23)	(-0.30)
Payment	0.024*	0.026*	0.029*	0.028
	(1.73)	(1.72)	(1.72)	(1.41)
Non-cross	0.003	-0.001	0.008	0.006
	(0.40)	(-0.14)	(0.74)	(0.52)
Non-related	-0.007	-0.004	-0.004	0.003
	(-0.72)	(-0.39)	(-0.36)	(0.20)
Major	0.003	-0.004	0.014	0.010
	(0.19)	(-0.26)	(0.79)	(0.50)
Price	0.005	0.003	0.005	0.001
	(1.54)	(1.01)	(1.29)	(0.25)
2018 M&A year	0.012	0.020**	0.031***	0.024**
	(1.48)	(2.19)	(2.75)	(2.03)
2019 M&A year	0.025**	0.019*	-0.020	-0.026**
	(2.43)	(1.70)	(-1.61)	(-1.98)
Constant	-0.216***	-0.169**	-0.264***	-0.178*
	(-3.16)	(-2.17)	(-2.88)	(-1.76)
Industry FE	Yes	Yes	Yes	Yes
Observations	616	616	616	616
R-squared	0.094	0.092	0.159	0.128
Adjusted R-squared	0.064	0.061	0.131	0.098
F-statistics	3.209	2.850	6.248	4.109
Prob > F-statistics	0.000	0.000	0.000	0.000

Variations in the financial performance of mergers were calculated using CRS and VRS models. t-statistics are reported in parentheses

*, **, and ***, indicating significance at 10%, 5% and 1% levels, respectively.

Table 10 presents subsample robust regression results to evaluate all hypotheses in short-term after M&As. In columns (3), (5) and (6), for special equipment, computer communication and software information sectors based on CRS model, we found that Experience coefficients were 0.040, 0.027 and 0.033, and were significant at 10%, 5% and 10% levels, suggesting a positive correlation between Experience and post-M&A short-term financial performance variation. In columns (1), (2) and (5), for chemicals, pharmaceuticals and computer communication sectors based on CRS model, Debt coefficients were 0.095, 0.172 and 0.090, and were significant at 10%, 1% and 5% levels, presenting a positive correlation between Debt and post-M&A short-term financial performance variation. In columns (1) and (4), for chemicals and electrical machinery sectors based on CRS model, Type coefficients were 0.073 and 0.082, and were significant at 5% and 10% levels, suggesting a positive correlation between Type and post-M&A short-term financial performance variation. Similar results obtained from VRS model were also obtained within S1 Table; therefore, proposed hypotheses were supported for most sectors in short-term after M&As.

**Table 10:** Subsample robust regression results of Experience, Debt, Type on post-M&A short-term financial performance variation.

Variable	(1)	(2)	(3)	(4)	(5)	(6)
	ΔSTPcrs Chemicals	ΔSTPcrs Pharmaceuticals	ΔSTPcrs Special Equipment	ΔSTPcrs Electrical Machinery	ΔSTPcrs Computer Communication	ΔSTPcrs Software Information
Experience	-0.002	-0.047	0.040*	-0.008	0.027**	0.033*
	(-0.13)	(-1.60)	(1.91)	(-0.41)	(1.99)	(1.82)
Debt	0.095*	0.172***	0.103	-0.017	0.090**	0.038
	(1.95)	(2.85)	(1.44)	(-0.28)	(1.99)	(0.53)
Type	0.073**	-0.011	-0.017	0.082*	0.009	-0.032
	(2.56)	(-0.45)	(-0.36)	(1.97)	(0.36)	(-0.77)
SOE	0.004	0.044*	-0.032	0.038**	0.012	-0.031
	(0.27)	(1.78)	(-1.26)	(2.07)	(0.64)	(-1.50)
Top1	-0.006	0.023	0.193**	-0.048	0.165***	-0.024
	(-0.10)	(0.29)	(2.62)	(-0.94)	(3.72)	(-0.20)
PE	0.000	0.000	0.000	0.000**	0.000***	-0.000
	(0.52)	(1.41)	(1.32)	(2.46)	(3.50)	(-0.48)
Goal (vertical)	-0.003	-0.010	0.002	-0.016	-0.005	-0.019
	(-0.16)	(-0.30)	(0.06)	(-0.89)	(-0.21)	(-0.52)
Goal (others)	0.010	-0.006	0.019	-0.032	-0.004	0.005
	(0.50)	(-0.15)	(0.64)	(-1.43)	(-0.16)	(0.11)
Payment	0.011	-0.064	0.130***	-0.022	0.002	0.048
	(0.45)	(-0.94)	(3.69)	(-0.81)	(0.17)	(1.44)
Non-cross	-0.049***	0.008	-0.023	0.052***	-0.007	0.031
	(-2.65)	(0.36)	(-0.68)	(3.14)	(-0.50)	(1.57)
Non-related	-0.006	-0.037	-0.005	0.001	-0.033**	0.008
	(-0.35)	(-1.43)	(-0.17)	(0.03)	(-2.38)	(0.32)
Major	0.019	-0.026	0.093***	-0.016	-0.026	-0.003
	(0.83)	(-0.40)	(3.33)	(-0.50)	(-1.44)	(-0.09)
Price	0.005	-0.004	-0.001	0.005	-0.003	0.007
	(0.81)	(-0.61)	(-0.05)	(0.94)	(-0.57)	(0.83)
2018.M&A year	0.023	-0.002	-0.034	0.008	-0.009	0.073***
	(1.38)	(-0.08)	(-1.24)	(0.43)	(-0.64)	(3.01)
2019.M&A year	0.157***	-0.021	-0.028	0.018	-0.092***	0.039
	(9.13)	(-0.79)	(-0.96)	(0.90)	(-4.90)	(1.36)
Constant	-0.186	0.114	-0.211	-0.095	0.005	-0.244
	(-1.49)	(0.72)	(-0.84)	(-0.78)	(0.04)	(-1.43)
Observations	115	73	85	101	139	103
R-squared	0.588	0.310	0.296	0.227	0.399	0.203
Adjusted R-squared	0.525	0.128	0.143	0.091	0.326	0.065
F-statistics	15.630	1.762	4.558	1.577	6.434	2.063
Prob > F-statistics	0.000	0.064	0.000	0.097	0.000	0.019

Variations in the financial performance of mergers were calculated using CRS model. t-statistics are reported in parentheses

*, **, and ***, indicating significance at 10%, 5% and 1% levels, respectively.

Table 11 summarizes subsample robust regression results to explore all hypotheses in medium-term after M&As. The results closely resemble those presented in Table 10, with a nuanced difference being that a positive correlation between Experience and post-M&A medium-term financial performance variation of mergers in computer communication and software information sectors becomes insignificant. Conversely, a significant positive correlation arises between these two variables in chemicals sector. To elaborate, in column (1) and (3), for chemicals and special equipment sectors using CRS model, the coefficients for Experience associated with post-M&A medium-term financial performance variation were 0.039 and 0.092, suggesting significant positive correlations at 10% and 1% levels. In columns (1) and (2), for chemicals and pharmaceuticals sectors based on CRS model, the coefficients for Debt associated with post-M&A medium-term financial performance variation were 0.176 and 0.185, respectively, suggesting a significant positive correlation at 5% level. In column (1) and (4), for chemicals and electrical machinery sectors using CRS model, the coefficients for Type associated with post-M&A medium-term financial performance variation were 0.073 and 0.067, suggesting significant positive correlations at 10% and 5% levels. Subsample robust regression using VRS model also reported similar results within S2 Table; therefore, proposed hypotheses were supported for most sectors in medium-term after M&As.

**Table 11:** Subsample robust regression results of Experience, Debt, Type on post-M&A medium-term financial performance variation.

Variable	(1)	(2)	(3)	(4)	(5)	(6)
	ΔMTPcrs Chemicals	ΔMTPcrs Pharmaceuticals	ΔMTPcrs Special Equipment	ΔMTPcrs Electrical Machinery	ΔMTPcrs Computer Communication	ΔMTPcrs Software Information
Experience	0.039*	-0.060	0.092***	0.025	0.010	0.014
	(1.68)	(-1.40)	(3.06)	(1.05)	(0.49)	(0.51)
Debt	0.176**	0.185**	0.002	-0.051	0.078	0.085
	(2.55)	(2.16)	(0.02)	(-0.68)	(1.24)	(1.04)
Type	0.073*	-0.029	-0.040	0.067**	0.028	0.006
	(1.79)	(-0.46)	(-0.82)	(2.13)	(0.61)	(0.12)
SOE	-0.004	0.068**	-0.078***	0.026	0.019	0.018
	(-0.18)	(2.24)	(-2.65)	(0.93)	(0.71)	(0.68)
Top1	0.045	-0.047	0.355***	-0.077	0.133**	-0.110
	(0.55)	(-0.47)	(3.55)	(-1.13)	(2.07)	(-0.98)
PE	0.000	0.000	0.000	0.000***	0.000	-0.000
	(0.88)	(1.29)	(1.14)	(2.77)	(1.17)	(-0.71)
Goal (vertical)	-0.015	-0.012	0.036	-0.005	0.024	0.001
	(-0.46)	(-0.27)	(1.07)	(-0.21)	(0.49)	(0.02)
Goal (others)	0.013	-0.022	0.068*	-0.028	0.014	0.030
	(0.39)	(-0.42)	(1.94)	(-1.02)	(0.28)	(0.67)
Payment	0.010	-0.038	0.087**	-0.038	0.015	0.080*
	(0.28)	(-0.51)	(2.04)	(-1.07)	(0.44)	(1.92)
Non-cross	-0.050**	-0.002	0.015	0.067***	0.018	0.022
	(-2.17)	(-0.07)	(0.34)	(3.32)	(0.76)	(0.94)
Non-related	-0.027	-0.043	0.011	-0.022	0.002	-0.010
	(-1.34)	(-1.02)	(0.31)	(-0.90)	(0.07)	(-0.30)
Major	0.022	-0.012	0.075**	-0.046	0.005	-0.013
	(0.56)	(-0.17)	(2.28)	(-1.16)	(0.18)	(-0.32)
Price	0.009	-0.003	0.006	-0.003	0.003	0.009
	(1.26)	(-0.27)	(0.50)	(-0.37)	(0.39)	(0.94)
2018.M&A year	0.057**	-0.020	-0.051	0.019	0.002	0.115***
	(2.44)	(-0.47)	(-1.66)	(0.86)	(0.09)	(3.83)
2019.M&A year	0.017	-0.036	-0.160***	-0.007	-0.104***	0.063*
	(0.65)	(-0.85)	(-4.30)	(-0.26)	(-3.66)	(1.78)
Constant	-0.296*	0.033	-0.407	0.042	-0.181	-0.325
	(-1.84)	(0.14)	(-1.50)	(0.27)	(-1.10)	(-1.60)
Observations	115	73	85	101	139	103
R-squared	0.330	0.248	0.468	0.205	0.200	0.251
Adjusted R-squared	0.229	0.050	0.352	0.065	0.102	0.122
F-statistics	2.763	1.385	5.360	2.121	2.321	2.871
Prob > F-statistics	0.001	0.186	0.000	0.016	0.006	0.001

Variations in the financial performance of mergers were calculated using CRS model. t-statistics are reported in parentheses

*, **, and ***, indicating significance at 10%, 5% and 1% levels, respectively.

## 6 Robustness check

As stipulated by the Shenzhen and Shanghai Stock Exchanges’ listing rules, controlling shareholders are prohibited from transferring or entrusting their shares for three years since the IPO date [58,59]. Based on a three-year listing duration, we categorize listed companies into mature and non-mature. This distinction reflects firms’ capital operation expertise, such as M&As, within the Chinese market. Besides, highly leveraged companies may seek to prevent managerial misuse of cash flow or leverage tax shield benefits [47,48]. Regression analyses using CRS and VRS models have consistently demonstrated the influence of Experience and Debt on short- and medium-term post-M&A financial performance. In addition, we also considered the method of replacing indicators for robustness test. Specifically, for assessing a company’s capital operation experience, we substituted the original dummy variable Experience with the numerical value of listing duration. Similarly, for examining the free cash flow agency issue, we replaced the original financial leverage level with free cash flow, which reflects the amount of free cash flow the company possessed in the financial year prior to the M&A completion. Table 12 presents the robustness test results of Listing Duration and Free Cash Flow on post-M&A financial performance. The results indicate that Listing Duration significantly enhances post-M&A financial performance. This finding aligns with the benchmark regression analysis outcomes. Notably, although Free Cash Flow does not exhibit statistical significance using VRS model, it significantly improves post-M&A financial performance based on CRS model. This conclusion is supported by prior research, potentially due to the minimization of agency conflicts in emerging markets with concentrated ownership, enabling free cash flow to alleviate post-M&A financial distress [73].

**Table 12:** Robustness test results of Listing Duration, Free Cash Flow on post-M&A financial performance variation.

Variable	(1)	(2)	(3)	(4)
	ΔSTPcrs	ΔSTPvrs	ΔMTPcrs	ΔMTPvrs
Listing Duration	0.002**	0.003***	0.002**	0.003***
	(2.31)	(3.68)	(2.15)	(3.01)
Free Cash Flow	0.018**	0.005	0.018**	0.002
	(2.48)	(0.68)	(2.19)	(0.16)
Type	0.029	0.033**	0.034	0.048**
	(1.52)	(1.97)	(1.48)	(2.39)
SOE	0.001	0.011	-0.002	-0.003
	(0.11)	(1.14)	(-0.16)	(-0.20)
Top1	0.019	0.016	0.000	0.004
	(0.65)	(0.57)	(0.01)	(0.12)
PE	0.017***	0.006	0.024***	0.014*
	(2.93)	(0.98)	(3.34)	(1.80)
Goal (vertical)	-0.011	-0.016	-0.005	-0.002
	(-0.99)	(-1.35)	(-0.38)	(-0.14)
Goal (others)	-0.004	-0.013	0.005	-0.002
	(-0.31)	(-0.96)	(0.33)	(-0.10)
Payment	0.025*	0.028*	0.031*	0.030
	(1.78)	(1.82)	(1.79)	(1.52)
Non-cross	0.003	-0.001	0.007	0.006
	(0.33)	(-0.10)	(0.70)	(0.57)
Non-related	-0.009	-0.005	-0.007	0.000
	(-1.00)	(-0.54)	(-0.64)	(0.04)
Major	0.002	-0.006	0.011	0.006
	(0.10)	(-0.40)	(0.65)	(0.33)
Price	0.005	0.003	0.006	0.002
	(1.64)	(1.14)	(1.47)	(0.54)
2018 M&A year	0.011	0.018**	0.029**	0.022*
	(1.33)	(2.03)	(2.55)	(1.85)
2019 M&A year	0.022**	0.016	-0.023*	-0.028**
	(2.20)	(1.49)	(-1.83)	(-2.14)
Constant	-0.199***	-0.150*	-0.247***	-0.161
	(-2.90)	(-1.94)	(-2.64)	(-1.61)
Industry FE	Yes	Yes	Yes	Yes
Observations	616	616	616	616
R-squared	0.095	0.077	0.151	0.107
Adjusted R-squared	0.065	0.046	0.123	0.077
F-statistics	2.816	2.296	5.549	3.437
Prob > F-statistics	0.000	0.001	0.000	0.000

Variations in the financial performance of mergers were calculated using CRS and VRS models. t-statistics are reported in parentheses

*, **, and ***, indicating significance at 10%, 5% and 1% levels, respectively.

## 7 Conclusion

This research investigated the effects of M&A activities on corporate financial performance and determinants of M&A performance of mergers in China from 2017 to 2019 using DEA models to eradicate the impacts of business cycle fluctuations. Through comparative analyses in the first part, we found that M&As failed to improve corporate financial performance and often decreased the financial performance of mergers compared to corresponding non-mergers, especially in pharmaceuticals, electrical machinery, and software information sectors. Potential reasons for this phenomenon might be significant integration of China into the global value chain since its accession to the World Trade Organization. Abundance of low-cost labor in China has made the country internationally competitive in labor-intensive industries [14] and China’s historic forward in foreign trade has accelerated the modernization and industrialization of enterprises and enhanced the income levels of individuals and households [15]. In recent years, due to China’s low industrial concentration, declining labor force, rising wages and other factors, enterprises compete to expand through M&As in fierce competitive environments to increase market shares and achieve economies of scale [16,74]. Furthermore, M&As have historically been encouraged by Chinese policy as a tool to change competitive dynamics in the market. Particularly, M&As have been applied as a mechanism for restructuring SOEs by introducing mixed ownership to improve operational efficiency and promote innovation [75]. Due to the late initiation of M&A activity, Chinese M&A market is assumed to be highly immature, which may engender a spectrum of challenges [14]. Firstly, lack of M&A experience in enterprises often leads to suboptimal post-acquisition integration, thereby impeding the realization of anticipated synergistic effects [76]. Secondly, managerial hubris frequently leads to high-premium M&As, giving rise to excess M&A goodwill. Subsequent impairment of goodwill erodes corporate profits [52,77].

In the second part of regression analyses and robustness check, we found that there were significant positive relationships between mergers’ sufficient listing duration, high financial leverages, adequate free cash flow, asset as target, and post-M&A performance of mergers. Accordingly, our findings offer new evidence that, opposite to the free cash flow hypothesis, abundant free cash flow is beneficial for mergers in China. These results emphasized that lack of experience and managerial hubris serve as the primary underlying factors contributing to underperformance observed in the Chinese M&A market. Due to the relatively late development of China’s capital market, listed companies lack sufficient experience in capital operations. Therefore, government regulatory authorities should strengthen capital market training for directors, supervisors, and senior management of listed companies to enhance their professional capabilities and facilitate enterprise development. In addition, prior to engaging in M&As, companies ought to engage professional evaluation teams to conduct meticulous and prudent assessments of target value, in order to mitigate the risk of excessive M&A premiums arising from managerial hubris.

The interpretation of our findings should consider the limitations of this research. In performance comparison part, we applied DEA models to calculate the financial performance of mergers and non-mergers within each sector pre- and post-M&As. By contrasting the mean performance variations of these two groups, we aimed to evaluate the potential impacts of M&As on corporate financial performance. While this method was statistically sound, it might overlook particular variations of individual DMUs, potentially obscuring relevant information for informed decision-making. Therefore, it is recommended that future works conduct in-depth analyses of performance variations among individual DMUs across two groups of samples in a particular sector. In addition, in analyses of performance impact factors part, similar to some previous studies [78,79], We used the Ordinary Least Squares (OLS) regression to examine the impact of a set of contextual factors on the variations in corporate financial performance pre- and post-M&As. However, the financial performance scores of the DMUs calculated by DEA models were bounded, the reliability of the results by OLS regression might be undermined. Therefore, we suggest adopting truncated regression model with a double bootstrapping procedure to effectively address this issue in future works [80,81].

## Supporting information

S1 Data Input and output data are used for DEA models. (XLSX)

S2 Data Variable data are used for regression models. (XLSX)

S1 Fig Comparative analyses of performance scores between mergers and non-mergers in 2017. (TIF)

S2 Fig Comparative analyses of performance scores between mergers and non-mergers in 2018. (TIF)

S3 Fig Comparative analyses of performance scores between mergers and non-mergers in 2019. (TIF)

S1 Table Subsample robust regression results of Experience, Debt, Type on post-M&A short-term financial performance variation. (DOCX)

S2 Table Subsample robust regression results of Experience, Debt, Type on post-M&A medium-term financial performance variation. (DOCX)
