# Supplementary figures and images for: Impact of mergers and acquisitions on firms’ performance adjusted to business cycle fluctuations in China
Source: PLoS One. 2025 Jan 24;20(1):e0318024. doi: 10.1371/journal.pone.0318024 (PMC11760017; doi:10.1371/journal.pone.0318024)

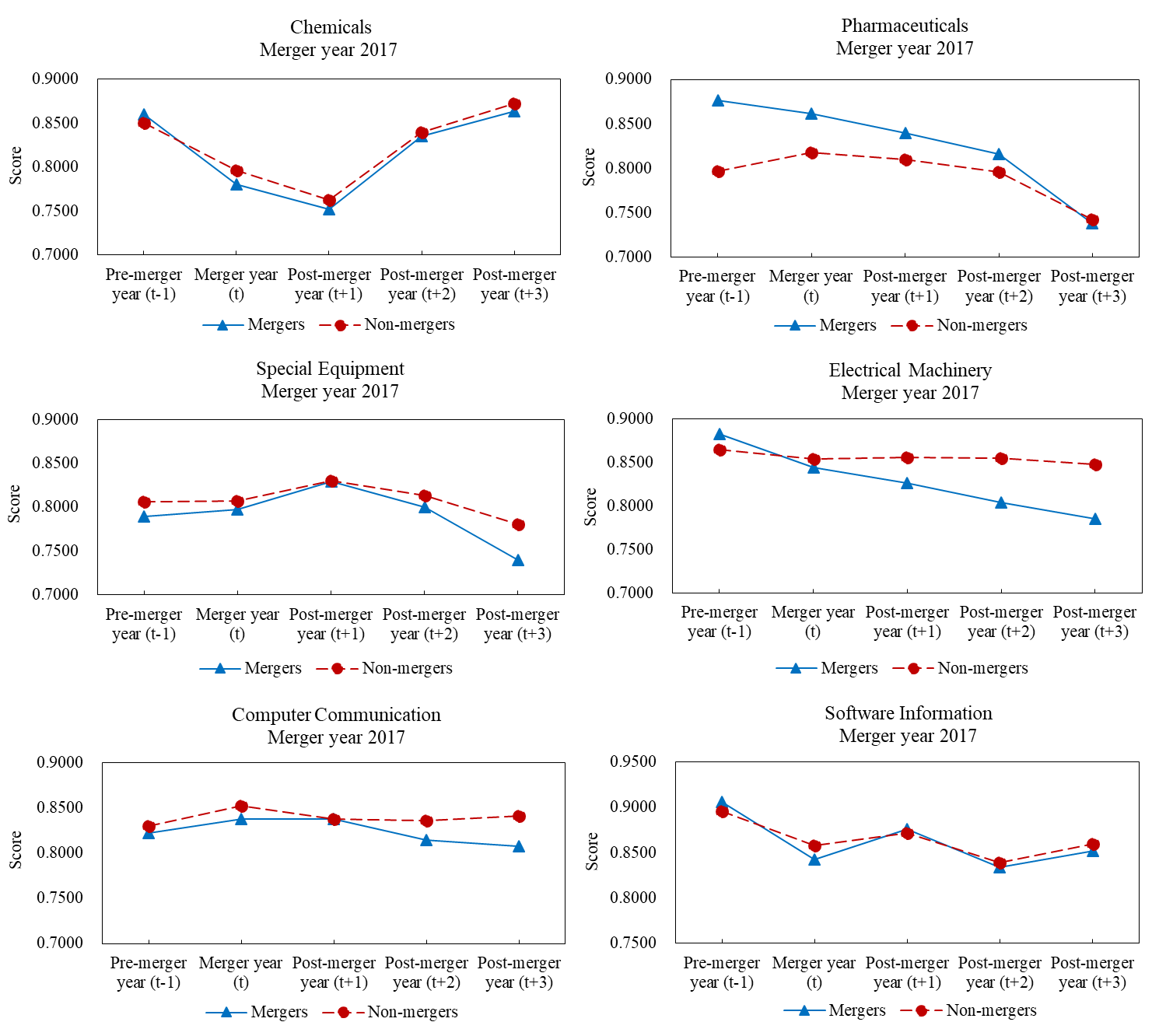

Supplement: S1 Fig — (TIF) [file pone.0318024.s003.tif]

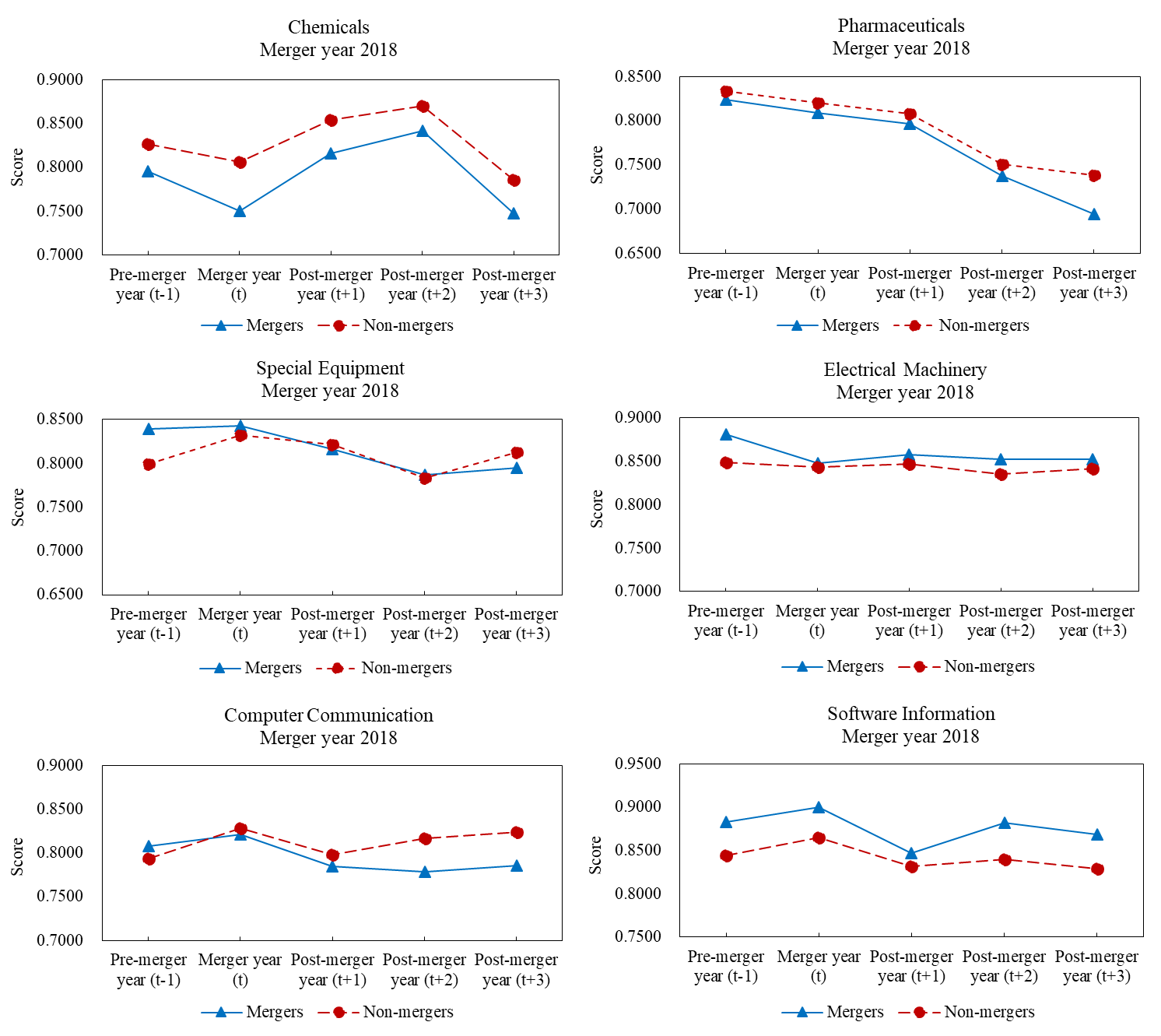

Supplement: S2 Fig — (TIF) [file pone.0318024.s004.tif]

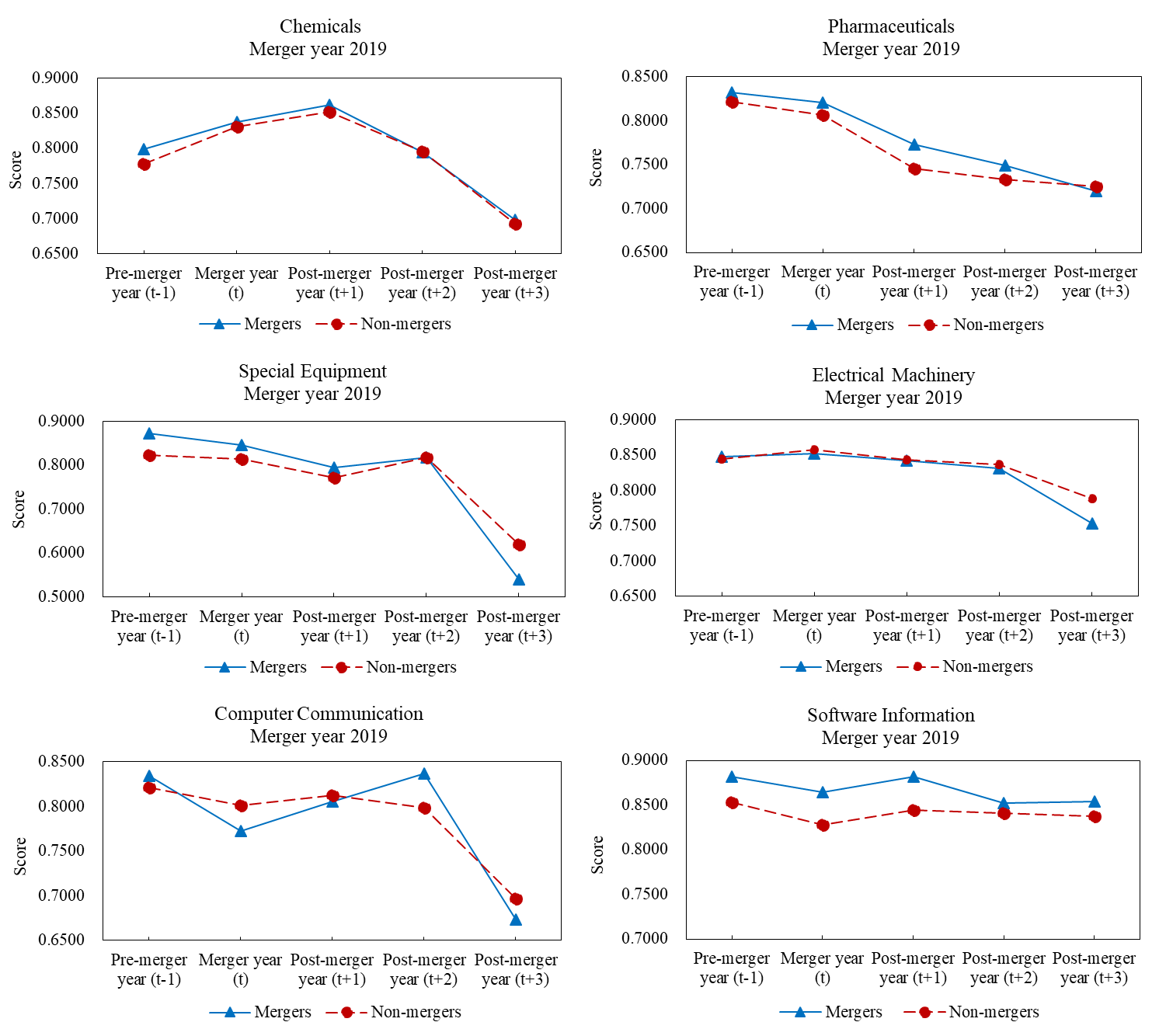

Supplement: S3 Fig — (TIF) [file pone.0318024.s005.tif]
