# Supplementary material for: Impact of mergers and acquisitions on firms’ performance adjusted to business cycle fluctuations in China
Source: PLoS One. 2025 Jan 24;20(1):e0318024. doi: 10.1371/journal.pone.0318024 (PMC11760017; doi:10.1371/journal.pone.0318024)
Supplement: S1 Table — (DOCX) [file pone.0318024.s006.docx]

**Table. Subsample robust regression results of Experience, Debt, Type on post-M&A short-term financial performance variation.**

| **Variable** | **(1)** | **(2)** | **(3)** | **(4)** | **(5)** | **(6)** |
| --- | --- | --- | --- | --- | --- | --- |
|  | **∆STPvrs**  **Chemicals** | **∆STPvrs**  **Pharmaceuticals** | **∆STPvrs**  **Special Equipment** | **∆STPvrs**  **Electrical Machinery** | **∆STPvrs**  **Computer Communication** | **∆STPvrs**  **Software Information** |
| Experience | 0.031 | 0.020 | 0.038* | 0.027 | 0.021 | 0.042** |
|  | (1.32) | (0.61) | (1.72) | (1.23) | (1.33) | (2.50) |
| Debt | 0.104* | 0.162** | -0.047 | 0.010 | 0.090* | 0.099 |
|  | (1.82) | (2.17) | (-0.67) | (0.14) | (1.68) | (1.60) |
| Type | 0.087** | -0.004 | -0.010 | 0.054* | 0.010 | -0.005 |
|  | (2.12) | (-0.13) | (-0.14) | (1.73) | (0.32) | (-0.22) |
| SOE | 0.054** | 0.027 | -0.009 | 0.027 | 0.023 | -0.039* |
|  | (2.36) | (0.99) | (-0.35) | (1.60) | (1.10) | (-1.83) |
| Top1 | 0.030 | 0.046 | 0.060 | -0.018 | 0.103* | 0.079 |
|  | (0.45) | (0.56) | (0.60) | (-0.32) | (1.93) | (0.86) |
| PE | 0.000 | 0.000 | 0.000 | 0.000 | 0.000 | -0.000 |
|  | (0.84) | (0.04) | (1.01) | (0.36) | (0.55) | (-0.37) |
| Goal (vertical) | -0.009 | -0.029 | -0.012 | -0.004 | -0.002 | -0.038 |
|  | (-0.27) | (-0.68) | (-0.38) | (-0.21) | (-0.05) | (-1.21) |
| Goal (others) | -0.011 | -0.022 | -0.024 | -0.014 | 0.010 | -0.003 |
|  | (-0.35) | (-0.49) | (-0.60) | (-0.58) | (0.31) | (-0.09) |
| Payment | 0.033 | 0.005 | 0.089** | -0.029 | -0.008 | 0.036 |
|  | (1.00) | (0.05) | (2.48) | (-1.18) | (-0.24) | (1.37) |
| Non-cross | -0.050* | 0.015 | -0.021 | 0.035* | -0.020 | 0.029 |
|  | (-1.98) | (0.63) | (-0.58) | (1.92) | (-1.10) | (1.61) |
| Non-related | -0.023 | -0.031 | 0.019 | 0.017 | -0.040** | 0.014 |
|  | (-1.07) | (-1.02) | (0.54) | (1.05) | (-2.10) | (0.49) |
| Major | 0.044 | -0.004 | 0.044 | -0.026 | -0.067** | 0.018 |
|  | (1.33) | (-0.05) | (1.31) | (-1.00) | (-2.04) | (0.66) |
| Price | 0.001 | -0.009 | 0.006 | -0.002 | -0.006 | 0.001 |
|  | (0.15) | (-1.07) | (0.67) | (-0.29) | (-0.96) | (0.12) |
| 2018.M&A year | 0.080*** | -0.004 | -0.028 | 0.024 | -0.013 | 0.033* |
|  | (3.38) | (-0.12) | (-0.96) | (1.54) | (-0.74) | (1.68) |
| 2019.M&A year | 0.131*** | -0.047 | -0.056 | 0.047** | -0.069*** | 0.024 |
|  | (5.67) | (-1.58) | (-1.59) | (2.06) | (-3.32) | (1.05) |
| Constant | -0.197 | 0.101 | -0.209 | -0.022 | 0.089 | -0.148 |
|  | (-1.29) | (0.52) | (-0.94) | (-0.17) | (0.65) | (-1.05) |
| Observations | 115 | 73 | 85 | 101 | 139 | 103 |
| R-squared | 0.430 | 0.186 | 0.235 | 0.181 | 0.243 | 0.232 |
| Adjusted R-squared | 0.344 | -0.028 | 0.069 | 0.036 | 0.151 | 0.100 |
| F-statistics | 6.363 | 1.102 | 1.646 | 2.917 | 3.739 | 2.529 |
| Prob > F-statistics | 0.000 | 0.376 | 0.084 | 0.001 | 0.000 | 0.004 |

Variations in the financial performance of mergers were calculated using VRS model. t-statistics are reported in parentheses; *, **, and ***, indicating significance at 10%, 5% and 1% levels, respectively.
