# Supplementary material for: Impact of mergers and acquisitions on firms’ performance adjusted to business cycle fluctuations in China
Source: PLoS One. 2025 Jan 24;20(1):e0318024. doi: 10.1371/journal.pone.0318024 (PMC11760017; doi:10.1371/journal.pone.0318024)
Supplement: S2 Table — (DOCX) [file pone.0318024.s007.docx]

**Table. Subsample robust regression results of Experience, Debt, Type on post-M&A medium-term financial performance variation.**

| **Variable** | **(1)** | **(2)** | **(3)** | **(4)** | **(5)** | **(6)** |
| --- | --- | --- | --- | --- | --- | --- |
|  | **∆MTPvrs**  **Chemicals** | **∆MTPvrs**  **Pharmaceuticals** | **∆MTPvrs Special Equipment** | **∆MTPvrs Electrical Machinery** | **∆MTPvrs Computer Communication** | **∆MTPvrs**  **Software Information** |
| Experience | 0.062** | -0.002 | 0.059** | 0.060** | 0.020 | 0.031 |
|  | (2.19) | (-0.03) | (2.05) | (2.13) | (0.94) | (1.18) |
| Debt | 0.166** | 0.182* | -0.135 | -0.028 | 0.067 | 0.191** |
|  | (2.20) | (1.93) | (-1.34) | (-0.30) | (1.01) | (2.55) |
| Type | 0.123** | -0.022 | -0.003 | 0.020 | 0.049 | 0.026 |
|  | (2.46) | (-0.41) | (-0.03) | (0.99) | (1.20) | (0.68) |
| SOE | 0.033 | 0.019 | -0.075** | 0.002 | 0.020 | 0.010 |
|  | (1.32) | (0.56) | (-2.20) | (0.06) | (0.74) | (0.38) |
| Top1 | 0.060 | -0.051 | 0.217* | -0.040 | 0.116* | -0.072 |
|  | (0.66) | (-0.51) | (1.87) | (-0.51) | (1.72) | (-0.64) |
| PE | 0.000 | 0.000 | 0.000 | 0.000 | -0.000 | -0.000 |
|  | (1.18) | (1.22) | (0.04) | (0.53) | (-0.39) | (-0.13) |
| Goal (vertical) | -0.008 | -0.016 | 0.033 | 0.016 | 0.022 | -0.021 |
|  | (-0.20) | (-0.34) | (0.86) | (0.56) | (0.42) | (-0.54) |
| Goal (others) | -0.006 | -0.030 | 0.030 | 0.003 | 0.026 | 0.012 |
|  | (-0.14) | (-0.57) | (0.64) | (0.08) | (0.48) | (0.26) |
| Payment | 0.012 | 0.074 | 0.023 | -0.073 | -0.014 | 0.072* |
|  | (0.28) | (0.78) | (0.51) | (-1.46) | (-0.32) | (1.84) |
| Non-cross | -0.070** | 0.002 | -0.009 | 0.045* | 0.011 | 0.046** |
|  | (-2.52) | (0.06) | (-0.20) | (1.95) | (0.46) | (2.02) |
| Non-related | -0.036 | -0.035 | 0.046 | 0.005 | -0.010 | -0.002 |
|  | (-1.48) | (-0.76) | (1.11) | (0.20) | (-0.33) | (-0.05) |
| Major | 0.053 | 0.034 | 0.006 | -0.080* | -0.043 | 0.001 |
|  | (1.27) | (0.40) | (0.15) | (-1.67) | (-1.10) | (0.03) |
| Price | -0.001 | -0.004 | 0.009 | -0.011 | -0.004 | 0.006 |
|  | (-0.10) | (-0.32) | (0.75) | (-1.19) | (-0.48) | (0.68) |
| 2018.M&A year | 0.010 | -0.010 | -0.029 | 0.069*** | -0.003 | 0.064** |
|  | (0.38) | (-0.21) | (-0.86) | (2.97) | (-0.11) | (2.50) |
| 2019.M&A year | -0.054* | -0.023 | -0.151*** | 0.041 | -0.070*** | 0.035 |
|  | (-1.88) | (-0.51) | (-3.72) | (1.36) | (-2.67) | (1.00) |
| Constant | -0.106 | -0.101 | -0.311 | 0.151 | -0.019 | -0.291 |
|  | (-0.60) | (-0.39) | (-1.13) | (0.78) | (-0.11) | (-1.53) |
| Observations | 115 | 73 | 85 | 101 | 139 | 103 |
| R-squared | 0.294 | 0.164 | 0.405 | 0.233 | 0.122 | 0.251 |
| Adjusted R-squared | 0.187 | -0.056 | 0.276 | 0.098 | 0.015 | 0.122 |
| F-statistics | 2.453 | 1.054 | 4.651 | 6.322 | 1.850 | 2.945 |
| Prob > F-statistics | 0.004 | 0.418 | 0.000 | 0.000 | 0.035 | 0.001 |

Variations in the financial performance of mergers were calculated using VRS model. t-statistics are reported in parentheses; *, **, and ***, indicating significance at 10%, 5% and 1% levels, respectively.
